# Glucose control during pregnancy in patients with type 1 diabetes correlates with fetal hemodynamics: a prospective longitudinal study

**DOI:** 10.1186/s12884-024-06462-7

**Published:** 2024-04-11

**Authors:** Patrik Simjak, Katerina Anderlova, Dagmar Smetanová, Michal Kršek, Miloš Mráz, Martin Haluzík

**Affiliations:** 1 Clinic of Gynecology, Obstetrics and Neonatology, First Faculty of Medicine, Charles University and General University Hospital, Prague, Czech Republic; 2 Gennet s.r.o, Fetal Medicine Center, Prague, Czech Republic; 3 3rd Internal Clinic, First Faculty of Medicine, Charles University and General University Hospital, Prague, Czech Republic; 4 Diabetes Centre, Institute for Clinical and Experimental Medicine, Prague, Czech Republic

**Keywords:** Diabetes, Fetal hemodynamics, Glucose control, Echocardiography, Hypoglycemia, Hyperglycemia

## Abstract

**Background:**

Maternal diabetes adversely affects fetal cardiovascular system development. Previous studies have reported that the fetuses of mothers with diabetes exhibit both structural and functional changes; nevertheless, prior studies have not examined the association between glucose control and fetal cardiac morphology and performance. Thus, the objective was to determine the association between fetal cardiac morphology and function and maternal glucose control in type 1 diabetes and to compare the differences in measured cardiac parameters between the fetuses of mothers with diabetes and healthy controls.

**Methods:**

In this prospective, longitudinal case-control study — including 62 pregnant women with type 1 diabetes mellitus and 30 healthy pregnant women — fetal cardiac assessment using B-mode, M-mode, and spectral pulsed-wave Doppler was performed in the second and third trimesters. In women with T1DM, glycated hemoglobin and data obtained from glucose sensors — including the percentage of time in, below, and above the range (TIR, TBR, and TAR, respectively), and coefficient of variation (CV) — were analyzed across three time periods: the last menstrual period to 13 (V1), 14–22 (V2), and 23–32 weeks (V3) of gestation. Fetal cardiac indices were compared between groups, and the correlation between glucose control and fetal cardiac indices was assessed.

**Results:**

At 28–32 weeks, the fetuses of women with T1DM exhibited increased left ventricular end-diastolic length, relative interventricular septum thickness, right ventricular cardiac output, and pulmonary valve peak systolic velocity compared with healthy controls. At 18–22 weeks, pulmonary and aortic valve diameters, left and right ventricular stroke volumes, and left cardiac output inversely correlated with the CV and glycated hemoglobin levels at V1 and V2. Furthermore, at 28–32 weeks, pulmonary and aortic valve diameters, left ventricular stroke volume, cardiac output, and right/left atrioventricular valve ratio inversely correlated with the TBR at V1, V2, and V3. Moreover, diastolic functional parameters correlated with the TAR and glycated hemoglobin levels, particularly after the first trimester.

**Conclusion:**

In women with T1DM, maternal hyperglycemia during pregnancy correlates with fetal diastolic function, whereas glucose variability and hypoglycemia inversely correlate with fetal left ventricular systolic function in the second and third trimesters.

**Supplementary Information:**

The online version contains supplementary material available at 10.1186/s12884-024-06462-7.

## Key message

This prospective longitudinal study demonstrates that in women with T1DM, maternal hyperglycemia during pregnancy correlates with fetal diastolic function. In contrast, glucose variability and hypoglycemia inversely correlate with fetal left ventricular systolic function in the second and third trimesters.

**Supplementary Information:**

The online version contains supplementary material available at 10.1186/s12884-024-06462-7.

## Background

Type 1 diabetes mellitus (T1DM) is a chronic metabolic disease caused by absolute insulin deficiency due to the autoimmune destruction of pancreatic B-cells. Usually diagnosed in childhood or early adulthood, T1DM inevitably affects reproduction. The reported prevalence of T1DM in pregnancy is 4.1–4.7 per 1000 pregnancies [1, 2]. Maternal diabetes adversely affects fetal development in various ways, such as by significantly affecting the fetal cardiovascular system. Previous studies have reported that the fetuses of mothers with diabetes have more globular hearts, increased right and left sphericity indices, and subclinical systolic dysfunction in the second half of pregnancy [3, 4]; fetal hyperinsulinemia owing to enhanced maternal-fetal glucose transport is thought to be the underlying cause. Nevertheless, prior studies have not examined the association between glucose control, and fetal cardiovascular morphology and performance. Additionally, T1DM and gestational diabetes (GDM) were often combined, regardless of the differences in etiopathogenesis and clinical presentation.

Glucose sensors have been used in the care of pregnant women with T1DM for several years. This creates an opportunity for the noninvasive, instant monitoring of glycemia at any time during the day, thereby enabling early treatment decisions. These wearables perform real-time continuous glucose monitoring (CGM) to alert the user when preset glucose targets are exceeded or flash glucose monitoring (FGM) that indicates glycemia when a reader is applied to the sensor. While recent studies have confirmed the safety and accuracy of both methods during pregnancy [5, 6], CGM is associated with improved neonatal outcomes, presumably due to better diabetes compensation [7].

The adoption of glucose sensors allowed for the development of core metrics for understanding glycemic status — including the time in, below, and above the range (TIR, TBR, and TAR, respectively) — and the coefficient of variation (CV), which describe the effectiveness of treatment in more detail than the traditionally used glycated hemoglobin (A1c). Notably, the high sensitivity of the sensors also enables the detection of glycemic fluctuations in pregnant women with a negative oral glucose tolerance test [8]. Thus, these sensors provide an entirely new opportunity to study the relationship between diabetes compensation and the development of pregnancy-related complications.

The primary objective of this prospective study was to determine the association between fetal cardiac morphology and function and maternal glucose control in T1DM. Secondarily, we aimed to compare the differences in measured cardiac parameters between the fetuses of mothers with T1DM and healthy controls.

## Subjects and methods

### Study population

This prospective, longitudinal case-control study included 64 consecutively recruited pregnant women with T1DM, and 32 matched healthy pregnant women. Pregnant women attending the combined first-trimester screening between April 2018 and December 2022 were recruited. The general exclusion criteria were multiple pregnancies, and fetal structural or chromosomal abnormalities diagnosed during the pregnancy. Women assigned to the control group had a standard 75 g oral glucose tolerance test performed between 24 and 28 weeks of gestation. Two women met the American Diabetes Association (ADA) criteria for diagnosis of GDM and were excluded from the analysis. All participants provided written informed consent for all study procedures, and the study protocol was approved by the Human Ethics Review Board.

Information on the maternal body mass index (BMI), age, race, method of conception (natural or assisted by *in-vitro* fertilization), cigarette smoking during pregnancy, and parity were recorded at the first visit. In women with T1DM, the disease duration (in completed years) was calculated, and diabetes-related morbidity, sensor type, and treatment modalities were recorded.

In all women with T1DM, glucose monitoring was initiated either before pregnancy or during the first trimester; a FGM system (FreeStyle® Libre™; Abbott, Inc.) or glucose sensors (G6®; DexCom, Inc., San Diego, CA, USA; or Guardian™ 4; Medtronic, Inc., Minneapolis, MN, USA) for real-time CGM of the interstitial fluid were used. Women with diabetes were followed-up every 4 weeks; if necessary, the diabetologist adjusted the treatment to ensure optimal disease control. The data obtained from sensors — including the TIR (time spent in target [3.5–7.8 mmol/l]), TBR (time spent below target [< 3.5 mmol/l]), TAR (time spent above target [> 7.8 mmol/l]) percentages — A1c, and CV were analyzed across three time periods: the last menstrual period to 13 weeks (V1); 14–22 weeks (V2); and 23–32 weeks (V3) of gestation. Insulin was administered via an insulin pump or multiple daily injections.

Standard perinatal outcomes were recorded after delivery, including gestational age, mode of delivery, birthweight, the incidence of preeclampsia, neonatal hypoglycemia, congenital malformations, NICU admission and umbilical artery pH.

### Echocardiography and ultrasound assessment

In all women, fetal B-mode, M-mode, and spectral pulsed-wave (PW) Doppler examinations were performed in the second and early third trimesters (18–22 weeks and 28–32 weeks of gestation, respectively) as a part of the routine prenatal ultrasound examinations. One investigator (P.S.) performed all fetal ultrasound examinations using a VolusonTM E10 BT 18 ultrasound system (GE Healthcare, Chicago, Il, USA). All measurements were performed using a convex-array obstetric transducer (C2-9). M-mode was used to assess ventricular free and septal wall thicknesses, and chamber dimensions. An apical, basal, or lateral four-chamber view in B-mode was used to obtain measurements regarding the cardiac axis, cardiothoracic index, ventricular length, and ventricular and semilunar valve dimensions, as appropriate. The ventricular sphericity index was calculated as the ventricular end-diastolic diameter/end-diastolic length. The relative wall thicknesses of the ventricles and interventricular septum (IVS) were estimated as (2 × free wall or septal wall thickness)/ventricular end-diastolic diameter. PW Doppler with an angle correction of < 45° was used to obtain Doppler signals from the inflow and outflow tracts for the evaluation of diastolic and systolic function. The left ventricular (LV) myocardial performance index (MPI) was obtained from a single cardiac cycle by placing the sample volume at the junction of the anterior mitral valve leaflet and left outflow tract to simultaneously display ventricular filling and emptying. For the right ventricular (RV) MPI, inflow and outflow pulsed Doppler signals were obtained separately, and MPI was only calculated if the difference between the fetal heart rate in the inflow and outflow tracts was < 5 beats per minute. Fetal biometry was performed in all women, and the pulsatility indices of the uterine and umbilical arteries were assessed.

### Intra- and interobserver reproducibility

The same investigator (P.S.) repeated the measurements on 20 randomly selected fetal echocardiograms obtained in the second and third trimesters in the same cardiac cycle. A second observer (D.S.) repeated the measurements using the same echocardiogram. Intraclass correlation coefficients (ICCs) were calculated to assess intra- and interobserver variability.

### Statistical analysis

Sample size and power calculations were performed based on the assumption that in the second trimester, the mean septal thickness increases by 10% in the fetuses of women with poorly controlled diabetes compared with controls [9]. Assuming a standard deviation of 10%, with an alpha of 0.05 and a power of 85%, the required sample size should be 39 with an enrollment ratio of 2:1. The enrollment ratio in favor of women with T1DM was chosen with the primary outcome of the study in mind and accounted for the lower propensity of healthy pregnant women to comply with the study protocol.

The Shapiro-Wilk test was performed to assess the data distribution for normality. Normally distributed continuous variables were presented as mean ± SD, and nonnormally distributed variables as median (interquartile range). Nominal variables were presented as numbers (percentages). Maternal and fetal parameters were compared between groups using the independent-samples Student’s *t*-test or Mann-Whitney U-test for continuous variables, and Chi-squared test for categorical variables. The Friedman test was used to detect differences in diabetes compensation across the three time periods. Pearson’s correlation test was used to assess the relationship between diabetes compensation and fetal cardiac indices.

## Results

### Pregnancy characteristics and diabetes control

In total, 94 pregnant women consented to participate in the study, including 64 with T1DM and 32 healthy controls; two women from the T1DM group were excluded from the analysis due to fetal anomalies (common arterial trunk and caudal regression syndrome); two women in the healthy control group met the American Diabetes Association (ADA) criteria for diagnosis of GDM and were excluded. No significant differences in the baseline population characteristics were observed between the groups, excluding a higher BMI in women with T1DM. As expected, birthweight was higher, and cesarean section, preeclampsia, and neonatal hypoglycemia were more frequent in the T1DM group.

The demographic characteristics and perinatal outcomes of the enrolled women are summarized in Table 1. Of the 62 women with T1DM, 52 started using glucose sensors before conception, whereas the remaining started in the first trimester. CGM and FGM were performed in 44 and 18 women, respectively. Insulin was administered via insulin pump to 33 (53.2%) women, whereas 29 (46.8%) received multiple daily injections. Characteristics of women with type 1 diabetes mellitus are summarized in Table 2. In women with T1DM, the mean A1c levels gradually decreased from preconception to V2, and then increased at V3. The same pattern was observed in the control group, but A1c was significantly lower in all visits. As expected, in women T1DM, A1c correlated with the TAR and CV during pregnancy. By contrast, A1c inversely correlated with the TIR. Furthermore, an inverse correlation was observed between the TBR at V1 (Table S1). Compared with the first trimester, women spent more TIR and less TAR at subsequent intervals. The glucose control of women in both study groups is summarized in Table 3. The ADA criterion for good glucose control, a TIR > 70%, was achieved in 41% of women with diabetes at all follow-up periods.

**Table 1:** Demographic characteristics and pregnancy outcomes of the study population

	T1DM (*n* = 62)	Controls (*n* = 30)
*Maternal and pregnancy characteristics*		
Age (years)	31.4 ± 3.8	32.5 ± 4.8
Caucasian ethnicity	62 (100)	30 (100)
BMI at enrolment (kg/m^2^)	23.8 (22.2–26.0)^†^	21.4 (19.5–22.8)
Nulliparity	32 (51.6)	19 (63.3)
IVF	4 (6.5)	5 (16.7)
Cigarette smoking	4 (6.5)	0
*Pregnancy outcomes*		
Gestational age at delivery (wk)	38 (37–39) ^†^	39 (38–40)
Cesarean section	41 (66.1) ^†^	11 (36.7)
Preeclampsia	5 (8.1) ^†^	0
Birth weight	3700 (3300–4090)*	3380 (3300–3567)
Umbilical artery pH	7.25 (7.17–7.31)	7.29 (7.19–7.34)
Neonatal hypoglycemia	28 (45.2) ^†^	1 (3.3)
NICU admission	10 (16.1)	2 (6.7)

Data are given as n (%), mean ± SD, or median (interquartile range)

^*^P < 0.05; ^†^*P* < 0.01

BMI: body mass index; IVF: in vitro fertilization; NICU: neonatal intensive care unit

**Table 2:** Characteristics of women with type 1 diabetes mellitus

	T1DM (*n* = 62)
T1DM duration [years]	14.0 ± 7.4
Retinopathy	51 (82.3)
Nephropathy	4 (6.5)
Neuropathy	1 (1.6)
Glucose sensor since preconception	52 (83.9)
Continuous glucose monitoring	44 (71.0)
Flash glucose monitoring	18 (29.0)
Insulin pump	33 (53.2)
A1c prior conception [%]	6.5 (6.0–7.5)

Data are given as n (%), mean ± SD, or median (interquartile range)

A1c: glycated hemoglobin

**Table 3:** Glycemic control

	V1 (<14 weeks)		V2 (14–22 weeks)	V3 (23–32 weeks)
*Controls (n = 30)*				
A1c [%]	5.1 (5.0-5.2)^§^		4.7 (4.6–4.9)^*§^	5.0 (4.8–5.1)^†§^
*T1DM (n = 62)*				
A1c [%]	6.3 (5.6–6.5)^‡^	5.6 (5.3–6.0)^*‡^		5.8 (5.5–6.2)^*†‡^
TBR (< 3.5 mmol/L) [%]	4 (1–6)	4 (2–6)		4 (2–6)
TIR (3.5–7.8 mmol/L) [%]	70 (55–80)	71 (63–80)^*^		71 (64–81)^*^
TAR (> 7.8 mmol/L) [%]	25 (15–41)	23 (12–33)^*^		24 (13–33)^*^
CV [%]	31.6 (29.9–36.6)	31.1 (28.4–34.8)		30.3 (27.6–33.0)^*^

Data are given median (interquartile range)

^*^P < 0.05 vs. <14 weeks; ^†^*P* < 0.05 vs. 14–22 weeks; ^‡^*P* < 0.05 prior to conception; ^§^ < 0.05 vs. T1DM

A1c: glycated hemoglobin; TBR: time below range; TIR: time in range; TAR: time above range; CV: coefficient of variation

### Cardiac geometry and function

The groups were comparable regarding the estimated fetal weight and pulsatility indices in the umbilical and uterine arteries. No differences in fetal cardiac geometry and function were observed between the groups at 18–22 weeks. At 28–32 weeks, the fetuses of women with T1DM exhibited increased LV end-diastolic length, relative IVS thickness, RV cardiac output (RV-CO), and pulmonary valve peak systolic velocity compared with the fetuses of healthy controls. A summary of the cardiac parameters in women with T1DM and controls in the second and third trimesters is presented in Table 4. The ICCs indicated moderate to excellent (ICC: 0.60–0.99) intra- and interobserver reliabilities (Table S2).

**Table 4:** Cardiac geometry and function in the fetuses of mothers with diabetes and healthy controls

Parameter	18–22 weeks		28–32 weeks	
	T1DM	Controls	T1DM	Controls
EFW (Hadlock)	415 (371–455)	415 (378–456)	1737 (1565–1941)	1691 (1551–1853)
Umbilical artery PI	1.09 (0.97–1.22)	1.09 (1.01–1.16)	1.00 (0.86–1.07)	0.93 (0.82–1.05)
Mean uterine artery PI	0.83 (0.71–1.01)	0.96 (0.90–1.20)	0.64 (0.54–0.77)	0.75 (0.60–0.81)
*Cardiac geometry*				
Cardiothoracic ratio (area)	0.34 ± 0.05	0.35 ± 0.04	0.43 ± 0.06	0.41 ± 0.05
Heart area (mm^2^)	422 ± 79	430 ± 84	1273 ± 204	1138 ± 146^*^
Thoracic area (mm^2^)	1244 (1097–1311)	1226 (1113–1369)	3036 (2728–3292)	2805 (2628–3088)
LV-EDD (mm)	6.24 ± 1.25	6.63 ± 1.34	10.60 ± 2.00	11.10 ± 1.88
RV-EDD (mm)	6.44 ± 1.30	6.31 ± 1.21	11.51 ± 1.45	11.25 ± 2.02
RV-EDD/LV-EDD ratio	1.06 ± 0.23	0.98 ± 0.22	1.09 (0.90–1.27)	1.06 (0.89–1.17)
LV-EDL (mm)	13.7 (12.6–15.1)	13.4 (12.8–14.6)	23.4 (21.3–24.9)	20.3 (19.7–22.2)^*^
RV-EDL (mm)	11.4 (10.0–12.7)	11.5 (10.5–12.7)	19.8 (17.9–21.8)	17.8 (16.5–19.4)
RAVV/LAVV ratio	1.00 ± 0.12	0.96 ± 0.12	1.07 ± 0.14	1.03 ± 0.11
LV sphericity index	0.45 (0.39–0.54)	0.47 (0.41–0.57)	0.44 (0.38–0.52)	0.52 (0.47–0.58)
RV sphericity index	0.57 (0.49–0.67)	0.54 (0.50–0.58)	0.58 (0.51–0.65)	0.61 (0.51–0.71)
Relative LV wall thickness	0.64 (0.50–0.86)	0.48 (0.43–0.70)	0.60 (0.44–0.84)	0.49 (0.37–0.68)
Relative RV wall thickness	0.67 (0.52–0.91)	0.54 (0.42–0.81)	0.59 (0.45–0.68)	0.50 (0.39–0.75)
Relative IVS thickness	0.68 ± 0.28	0.65 ± 0.28	0.71 ± 0.26	0.60 ± 0.20*
AV diameter (mm)	3.70 (3.32–4.01)	3.70 (3.45–4.30)	6.00 (5.70–6.70)	6.51 (5.40–6.95)
PV diameter (mm)	4.47 ± 0.51	4.54 ± 0.76	7.77 ± 0.94	7.67 ± 1.26
*Diastolic function*				
LV E (cm/s)	29.5 (27.5–32.9)	30.3 (26.0–32.7)	43.0 (35.6–47.0)	40.2 (36.7–45.7)
LV A (cm/s)	50.0 (44.7–54.7)	48.1 (41.9–52.2)	57.2 (50.5–65.1)	53.1 (45.7–60.4)
LV E/A	0.62 ± 0.08	0.64 ± 0.09	0.75 ± 0.11	0.78 ± 0.12
RV E (cm/s)	33.7 (30.5–38.2)	33.3 (30.6–40.0)	44.1 (38.3–49.2)	45.2 (40.5–50.3)
RV A (cm/s)	51.3 (47.5–57.1)	50.7 (46.4–56.9)	66.7 (52.5–69.5)	55.4 (51.3–61.3)
RV E/A	0.65 ± 0.09	0.68 ± 0.08	0.73 ± 0.10	0.77 ± 0.11
*Systolic function*				
FHR-LV (/min)	148 (143–152)	146 (143–151)	137 (132–146)	139 (133–145)
AV PSV (cm/s)	73.4 (64.0–79.4)	72.1 (64.1–79.7)	97.0 (86.4–109.2)	94.3 (82.6–100.3)
LV SV (mL)	0.88 (0.72–1.04)	0.85 (0.73–1.16)	3.20 (2.68–4.52)	3.26 (2.46–4.24)
LV-CO (mL/min/kg)	318 (247–388)	304 (268–400)	270 (225–332)	279 (206–340)
FHR-RV (/min)	146 (141–152)	148 (146–153)	139 (134–146)	143 (138–146)
PV PSV velocity (cm/s)	63.4 (54.6–74.0)	61.1 (55.8–69.5)	77.0 (67.8–100.0)	71.7 (66.8–82.2)^*^
RV SV (mL)	1.14 (0.95–1.33)	1.04 (0.91–1.41)	4.97 (3.29–5.86)	4.08 (3.04–5.17)^*^
RV-CO (mL/min/kg)	398 (348–461)	374 (322–480)	378 (301–474)	317 (276–440)
*Global myocardial performance*				
LV MPI	0.49 (0.43–0.56)	0.52 (0.42–0.57)	0.52 ± 0.09	0.53 ± 0.13
RV MPI	0.50 (0.44–0.59)	0.50 (0.44–0.56)	0.54 ± 0.08	0.53 ± 0.12

Data are given as mean ± SD, or median (interquartile range)

Compared with T1DM: ^*^P < 0.05, ^†^*P* < 0.01

EFW: estimated fetal weight; PI: pulsatility index; LV: left ventricle; RV: right ventricle; EDD: end-diastolic diameter; EDL: end-diastolic length; RAVV: right atrioventricular valve; LAVV left atrioventricular valve; IVS interventricular septum; E: early diastolic peak velocity; A: atrial contraction peak velocity; FHR: fetal heart rate; AV: aortic valve; SV: stroke volume; CO: cardiac output; PV: pulmonary valve; MPI: myocardial performance index

An inverse correlation between the CV and echocardiographic parameters was observed between 18 and 22 weeks. Specifically, pulmonary valve diameter and RV stroke volume (RV-SV) decreased with increasing CV prior to 14 weeks (*r* = − 0.51, *P* = 0.001; *r* = − 0.35, *P* = 0.033; respectively). Additionally, the CVs at V1 and V2 inversely correlated with the LV-SV (*r* = − 0.33, *P* = 0.043; *r* = − 0.41, *P* = 0.008; respectively), and aortic valve (AV) diameter and LV-CO at V2 (*r* = − 0.31, *P* = 0.046; *r* = − 0.39, *P* = 0.011; respectively). An inverse correlation was also observed between A1c and AV diameter at V1 (*r* = − 0.32, *P* = 0.018), and LV-SV and LV-CO at V1 and V2 (*r* = − 0.37, *P* = 0.006; *r* = − 0.27, *P* = 0.042; *r* = − 0.31, *P* = 0.024; *r* − 0.27, *P* = 0.041; respectively). Thus, increased glycemic variability and A1c in the first half of pregnancy impaired mid-gestation ventricular systolic function, and decreased semilunar valve diameters contributing to decreased SVs.

**Fig. 1:**
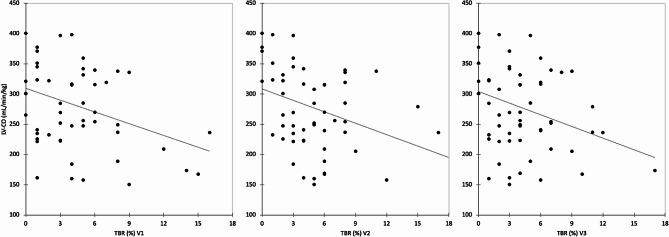
Scatterplots demonstrating the negative correlation between left ventricular cardiac output (LV-CO) and time below range (TBR) in the first 14 weeks (V1; *r* = -0.35, *P* = 0.023), at 14–22 weeks (V2; *r* = -0.34, *P* = 0.011), and at 23–28 weeks (V3; *r* = -0.29, *P* = 0.029) of gestation

At 28–32 weeks, the AV diameter inversely correlated with the TBR at V1, V2, and V3 (*r* = − 0.38, *P* = 0.006; *r* = − 0.35, *P* = 0.008; *r* = − 0.37, *P* = 0.005; respectively), but positively correlated with A1c at V3 (*r* = 0.30; *P* = 0.025). The TBR also inversely correlated with LV systolic function, represented by LV-SV (*r* = − 0.33, *P* = 0.029; *r* = − 0.32, *P* = 0.017; *r* = − 0.29, *P* = 0.024; for V1, V2, V3 respectively) and LV-CO (*r* = − 0.35, *P* = 0.023; *r* = − 0.34, *P* = 0.011; *r* = − 0.29, *P* = 0.029; for V1, V2, V3 respectively; Fig. 1). Moreover, LV-SV correlated with A1c at V3 (*r* = 0.27; *P* = 0.047).

By contrast, diastolic function markers correlated with A1c and TAR. At 28–32 weeks, the TAR correlated with RV A (*r* = 0.29, *P* = 0.009; *r* = 0.32, *P* = 0.017; *r* = 0.29, *P* = 0.047; for V1, V2, V3 respectively), LV E (*r* = 0.30, *P* = 0.034; *r* = 0.36, *P* = 0.008; for V2, V3 respectively), and LV A (*r* = 0.29, *P* = 0.031 for V3). Similarly, A1c correlated with RV E (*r* = 0.33, *P* = 0.014; *r* = 0.32, *P* = 0.020; for V2, V3 respectively), RV A (*r* = 0.30, *P* = 0.007; *r* = 0.36, *P* = 0.006; for V2, V3 respectively), and LV E (*r* = 0.28; *P* = 0.036; for V3). The correlations between glucose control and fetal cardiac geometry and function are summarized in Table 5.

**Table 5:** Correlations between diabetes compensation and fetal cardiac geometry and function

Variable	V1 (< 14 weeks)	V2 (14–22 weeks)	V3 (23–32 weeks)
	**Fetal echocardiography at 18–22 weeks**		
*Coefficient of variation*			
PV diameter	-0.51^†^	-	-
AV diameter	-	-0.31^*^	-
LV SV	-0.33^*^	-0.41^†^	-
LV CO/kg	-	-0.39^*^	-
RV SV	-0.35^*^	-	-
*A1c*			
AV diameter	-0.32^*^	-	-
LV SV	-0.37^†^	-0.27^*^	-
LV CO/kg	-0.31^*^	-0.27^*^	-
	**Fetal echocardiography at 28–32 weeks**		
*Time below range*			
AV diameter	-0.38^†^	-0.35^†^	-0.37^†^
LV SV	-0.33^*^	-0.32^*^	-0.29^*^
LV CO/kg	-0.35^*^	-0.34^*^	-0.29^*^
PV diameter	-0.30^*^	-	-0.28^*^
RAVV/LAVV ratio	-	-0.28^*^	-
*Time above range*			
RV A	0.29^*^	0.32^*^	0.35^†^
LV E	-	0.30^*^	0.36^†^
LV A	-	-	0.29^*^
*A1c*			
LV E	-	-	0.28^*^
RV E	-	0.33^*^	0.32^*^
RV A	-	0.36^†^	0.38^†^
AV diameter	-	-	0.30^*^
LV SV	-	-	0.27^*^

Pearson’s correlation coefficient (r) is presented

^*^P < 0.05, ^†^*P* < 0.01

PV: pulmonary valve; AV: aortic valve: LV: left ventricle; SV: stroke volume; CO: cardiac output; RV: right ventricle; RAVV: right atrioventricular valve; LAVV left atrioventricular valve; A1c: glycated hemoglobin; E: early diastolic peak velocity; A: atrial contraction peak velocity

## Discussion

This study presents a detailed analysis of fetal cardiovascular hemodynamics during the second and early third trimesters of patients with T1DM in relation to glucose control, and a comparison with healthy controls. The results demonstrate that in the study population, where 41% of women achieved the recommended glucose control target (> 70% of the TIR) throughout pregnancy, significant changes in cardiac geometry and function were only observed in the early third trimester. Additionally, this study demonstrates for the first time that glucose variability and maternal hypoglycemia affect LV performance in the second and third trimesters. By contrast, hyperglycemia during pregnancy affects the fetal diastolic function. Thus, optimal control of T1DM enhances fetal hemodynamics. However, the extent to which improvements in fetal hemodynamics can translate into improved clinical outcomes remains to be elucidated.

Although the pathophysiology of impaired fetal cardiac development in maternal diabetes is complex and incompletely understood, increased transplacental glucose transport and subsequent fetal hyperinsulinemia are thought to be the main causes [10]. Fetal hyperinsulinemia can alter placental mRNA expression, leading to the dysregulation of insulin/insulin-like growth factor (IGF) systems [11]. IGF-1 is a potent stimulator of cell growth, and experimental studies have demonstrated that it promotes prenatal cardiomyocyte growth [12]. Additionally, a positive correlation between cord blood IGF-1 bioavailability and IVS thickness was observed in the newborns of mothers with diabetes [13]. Other major factors that influence fetal heart morphology and function include increased oxidative stress, subclinical low-grade inflammation, maternal obesity, triglyceridemia, and placental dysfunction [13–15].

Similar to previously published studies, we demonstrated distinct changes in fetal cardiac morphology in the fetuses of mothers with T1DM during the third trimester, compared with controls. These included increased heart area, greater LV-EDL, and a thicker IVS [16]. Other studies have reported a more globular heart shape with increased ventricular sphericity indices [4, 17]; however, the cohorts in these studies mainly comprised women with gestational diabetes and fetal echocardiography was performed later in the third trimester, potentially explaining the noted discrepancies. A recent meta-analysis confirmed IVS thickening in the fetuses of women with T1DM; however, increased septal thickness has also been observed in the second trimester [16].

Previous research on pregnant women with diabetes has also revealed impaired fetal cardiac function, which can be demonstrated using various ultrasound examination techniques at different stages of pregnancy. The very first detectable manifestation of fetal heart function is the heart rate. One study found that pregnant women with pregestational diabetes (type 1 and 2) had a higher fetal heart rate in the first trimester compared to healthy women, regardless of their BMI [18]. However, our study found that the fetal heart rate was similar in both groups during the later trimesters.

Another parameter, fetal MPI, has been assessed by two recent meta-analyses concerning diabetes in pregnancy [16, 19]. In the study by Depla et al., fetal MPI in women with pregestational diabetes and controls were comparable, but Sirico et al. observed higher MPI in fetuses of diabetic mothers in the third trimester. Although both were published in a similar time period, each had different study inclusion criteria, which affected the results. Nevertheless, the MPI may be confounded by coexisting complications that are common in women with diabetes, such as maternal obesity, fetal macrosomia, or placental function. We did not observe differences in left or right ventricular MPI between the groups, possibly due to the comparable estimated fetal weight and uteroplacental Doppler indices between the groups or the limited number of study participants. Nevertheless, fetal MPI was unrelated to glucose control in our cohort.

Furthermore, we failed to demonstrate impaired diastolic function in the second and early third trimesters using spectral PW Doppler, which is consistent with the results of another published study [20]. However, a lower diastolic strain rate was observed in this study, suggesting that speckle-tracking echocardiography is a more sensitive method for assessing fetal heart dysfunction [20]. These subtle subclinical changes likely precede diastolic dysfunction, as demonstrated using conventional ultrasound parameters during the third trimester [21]. A similar impairment of diastolic function was also observed in the fetuses of mothers with gestational diabetes [22]; thus, hyperglycemia-induced cardiac remodeling and the consequent fetal adaptation may be responsible for this phenomenon. Indeed, we observed a positive correlation between the percentage of time spent in hyperglycemia, A1c, and diastolic function, mainly regarding the RV A wave. In line with our findings, a lower right E/A ratio was observed in the fetuses of women with poorly controlled pregestational diabetes [20, 23].

Regarding fetal systolic function in pregnancies complicated by T1DM, the evidence is inconclusive. A higher RV-SV was observed; however, the difference in CO was not significant after correcting for estimated fetal weight. A similar finding was observed at the end of the third trimester in a mixed cohort including the fetuses of mothers with GDM and T1DM [4]. In another study, RV systolic impairment was demonstrated using speckle-tracking echocardiography [24]. Although a difference in the LV-CO was not observed in the second and early third trimesters in the fetuses of women with T1DM, another study demonstrated a significant decrease in LV-CO at term [4]. In these fetuses, LV-CO was restored to values comparable to those in the healthy population early after birth, suggesting a suppressive effect of diabetes on heart function. A novel finding of our study was that LV-CO inversely correlated with glucose variability and A1c in the second trimester, and the percentage of time spent in hypoglycemia in the third trimester, independent of fetal weight.

As CO is dependent on the diameter of the corresponding semilunar valve and fetal heart rate an inverse correlation between the TBR and AV diameter contributed to this finding. Notably, up to 40% of women experience severe hypoglycemia during pregnancy [25]. Our finding that hypoglycemia impairs heart function is also supported by an earlier study that reported decreased fetal heart rate variability during maternal hypoglycemia episodes [26]. Nevertheless, maintaining a sufficient fetal CO is crucial to ensure adequate perfusion of the placenta, especially when fetal oxygen requirements peak in the late third trimester. Furthermore, women with T1DM exhibit increased placental angiogenesis, leading to a larger distribution volume and thus, a decreased afterload [27]. Strict compensation for diabetes with frequent episodes of hypoglycemia can lead to chronic hypoxia that may not manifest as overt fetal growth restriction, as the fetuses of pregnant women with diabetes are often predisposed to being macrosomic. We hypothesized that in most susceptible fetuses with diabetic fetopathy, this may even result in sudden intrauterine fetal demise in women with seemingly good compensation. Thus, with respect to strict glycemic control in pregnant women with T1DM, caution is necessary to prevent potentially harmful episodes of maternal hypoglycemia, especially in the third trimester.

## Conclusion

This is the first prospective longitudinal study to evaluate the association between fetal cardiac geometry and function and glucose control in a cohort of women with T1DM. It highlights the importance of optimal glucose control in women with T1DM during pregnancy, as maternal hyperglycemia during pregnancy correlates with fetal diastolic function, whereas glucose variability and hypoglycemia inversely correlate with fetal left ventricular systolic function in the second and third trimesters.

The main strength of this study was the prospective monitoring of glucose control using glucose sensors, and ultrasound evaluation of fetal cardiac function by a multidisciplinary team of specialists in diabetology and fetal medicine. Another strength was the consecutive recruitment of women with diabetes to minimize selection bias. During the study period, the estimated weight and routine Doppler indices of fetuses of women with diabetes did not differ from those of healthy controls; thus, we believe that these variables had negligible confounding effects on the presented results.

The main limitation of the present study was that the sample size was too small to allow for adjustment for potential confounders. The reason for the inclusion of fewer healthy controls was the lower willingness of women to comply with the study protocol, which required all ultrasound examinations and delivery at the investigating centre. Recruitment of healthy controls was also undermined by anti-epidemic measures during the SARS-CoV 19 pandemic.

The ultrasound examinations were performed by a single examiner who was not blinded to the diagnosis of diabetes, which implies the possibility of bias. Therefore, the measurements were repeated by the second blinded observer resulting in moderate to excellent interobserver agreements.

### Electronic supplementary material

Below is the link to the electronic supplementary material.

Supplementary Material 1

## Data Availability

The raw data can be obtained on request from the corresponding author.

## Abrreviations

A1c: Glycated hemoglobin
A: Atrial contraction peak velocity
AV: Aortic valve
CO: Cardiac output
CV: Coefficient of variation
E: Early diastolic peak velocity
EDD: End-diastolic diameter
EDL: End-diastolic length
EFW: Estimated fetal weight
IVS: Interventricular septum
LAVV: Left atrioventricular valve
LV: Left ventricle
MPI: Myocardial performance index
PI: Pulsatility index
PV: Pulmonary valve
RAVV: Right atrioventricular valve
RV: Right ventricle
SV: Stroke volume
T1DM: Type 1 diabetes
TAR: Time above range
TBR: Time below range
TIR: Time in range
