# Supplementary material for: Glucose control during pregnancy in patients with type 1 diabetes correlates with fetal hemodynamics: a prospective longitudinal study
Source: BMC Pregnancy Childbirth. 2024 Apr 11;24:264. doi: 10.1186/s12884-024-06462-7 (PMC11007889; doi:10.1186/s12884-024-06462-7)
Supplement: Supplementary file 1 — Supplementary Material 1 [file 12884_2024_6462_MOESM1_ESM.docx]

**Supplementary Material**

**Table S1.** Correlations between A1c and glucose sensor metrics

| *A1c* | V1 (<14 weeks) | V2 (14–22 weeks) | V3 (23–32 weeks) |
| --- | --- | --- | --- |
| TBR | -0.38^†^ | - | - |
| TIR | -0.69^†^ | -0.73^†^ | -0.64^†^ |
| TAR | 0.74^†^ | 0.75^†^ | 0.65^†^ |
| CV | 0.39^*^ | 0.48^†^ | 0.41^†^ |

Pearson correlation coefficient (r) is presented.

^*^P<0.05, ^†^P<0.01

A1c, glycated hemoglobin; TBR, time below range; TIR, time in range; TAR, time above range; CV, coefficient of variation

**Table S2.** Intra- and interobserver repeatability of fetal heart index measurements

| Measurements | Intraclass correlation coefficient | | | | |
| --- | --- | --- | --- | --- | --- |
|  | 18-22 Weeks | | 28-32 Weeks | 18-22 Weeks | 28-32 Weeks |
|  | ***Intraobserver variability N= 20*** | | | ***Interobserver variability N= 20*** | |
| Cardiothoracic ratio (area) | 0.75 | 0.62 | | 0.83 | 0.86 |
| Heart area (mm^2^) | 0.86 | 0.88 | | 0.87 | 0.92 |
| Thoracic area (mm^2^) | 0.96 | 0.86 | | 0.88 | 0.89 |
| LV-EDD | 0.98 | 0.97 | | 0.98 | 0.86 |
| RV-EDD | 0.96 | 0.99 | | 0.98 | 0.94 |
| RV/LV EDD | 0.93 | 0.98 | | 0.93 | 0.98 |
| LV-EDL | 0.66 | 0.72 | | 0.94 | 0.77 |
| RV-EDL | 0.73 | 0.84 | | 0.98 | 0.65 |
| LAVV | 0.83 | 0.93 | | 0.65 | 0.93 |
| RAVV | 0.79 | 0.78 | | 0.81 | 0.78 |
| RAVV/LAVV ratio | 0.67 | 0.93 | | 0.65 | 0.86 |
| LV sphericity index | 0.80 | 0.85 | | 0.83 | 0.60 |
| RV sphericity index | 0.87 | 0,95 | | 0.68 | 0.76 |
| LV wall thickness | 0.85 | 0.91 | | 0.78 | 0.81 |
| RV wall thickness | 0.83 | 0.82 | | 0.74 | 0.91 |
| IVS thickness | 0.88 | 0.61 | | 0.74 | 0.86 |
| Relative LV wall thickness | 0.84 | 0.89 | | 0.74 | 0.75 |
| Relative RV wall thickness | 0.80 | 0.95 | | 0.61 | 0.75 |
| Relative IVS thickness | 0.89 | 0.82 | | 0.76 | 0.62 |
| MV E | 0.95 | 0.96 | | 0.75 | 0.97 |
| MV A | 0.99 | 0.91 | | 0.90 | 0.99 |
| TV E | 0.98 | 0.84 | | 0.98 | 0.97 |
| TV A | 0.93 | 0.73 | | 0.84 | 0.97 |
| LV E/A | 0.94 | 0.88 | | 0.83 | 0.83 |
| RV E/A | 0.94 | 0.88 | | 0.98 | 0.84 |
| AV diameter | 0.85 | 0.87 | | 0.89 | 0.73 |
| Ao valve PS velocity | 0.99 | 0.97 | | 0.96 | 0.98 |
| FHR-LV | 0.99 | 0.99 | | 0.99 | 0.99 |
| SV left | 0.90 | 0.85 | | 0.90 | 0.83 |
| LV CO | 0.92 | 0.81 | | 0.92 | 0.78 |
| PV PS velocity | 0.89 | 0.88 | | 0.82 | 0.96 |
| FHR-RV | 0.97 | 0.98 | | 0.99 | 0.98 |
| PV diameter | 0.67 | 0.94 | | 0.80 | 0.92 |
| SV right | 0.59 | 0.97 | | 0.58 | 0.91 |
| RV CO | 0.62 | 0.96 | | 0.66 | 0.90 |
| LV MPI | 0.85 | 0.85 | | 0.69 | 0.77 |
| RV MPI | 0.95 | 0.96 | | 0.60 | 0.93 |

LV, left ventricle; RV, right ventricle; EDD, end-diastolic diameter; EDL, end-diastolic length; RAVV, right atrioventricular valve; LAVV, left atrioventricular valve; IVS, interventricular septum; Ao, aorta; FHR, fetal heart rate; SV, stroke volume; CO, cardiac output; PV, pulmonary valve; MPI, myocardial performance index; AV, aortic valve
